# Application of Medial Ganglionic Eminence Cell Transplantation in Diseases Associated With Interneuron Disorders

**DOI:** 10.3389/fncel.2022.939294

**Published:** 2022-07-05

**Authors:** Danping Li, Qiongfang Wu, Xiaohua Han

**Affiliations:** Department of Rehabilitation Medicine, Tongji Hospital, Tongji Medical College, Huazhong University of Science and Technology, Wuhan, China

**Keywords:** MGE cell, transplantation, GABAergic interneuron, epilepsy, schizophrenia, Alzheimer’s Disease, interneuron disorders/dysfunction, autism spectrum disorder

## Abstract

Excitatory projection neurons and inhibitory interneurons primarily accomplish the neural activity of the cerebral cortex, and an imbalance of excitatory-inhibitory neural networks may lead to neuropsychiatric diseases. Gamma-aminobutyric acid (GABA)ergic interneurons mediate inhibition, and the embryonic medial ganglionic eminence (MGE) is a source of GABAergic interneurons. After transplantation, MGE cells migrate to different brain regions, differentiate into multiple subtypes of GABAergic interneurons, integrate into host neural circuits, enhance synaptic inhibition, and have tremendous application value in diseases associated with interneuron disorders. In the current review, we describe the fate of MGE cells derived into specific interneurons and the related diseases caused by interneuron loss or dysfunction and explore the potential of MGE cell transplantation as a cell-based therapy for a variety of interneuron disorder-related diseases, such as epilepsy, schizophrenia, autism spectrum disorder, and Alzheimer’s disease.

## Introduction

Located in the ventral telencephalon of the embryo, the medial ganglionic eminence (MGE), the primordium of the globus pallidus, is a vital source of gamma-aminobutyric acid (GABA)ergic interneuron progenitors (Qi et al., 2018). During embryonic development, these cells migrate tangentially to the cortex, striatum, and hippocampal region of the brain to form inhibitory circuits (Marín and Rubenstein, 2001). The normal function of the cerebral cortex, including the neocortex, and hippocampus, requires a neural network consisting of glutamate projection neurons and GABAergic interneurons (Xu et al., 2004; Wonders and Anderson, 2006). GABAergic interneurons form inhibitory synapses in one or more parts of the brain and participate in the regulation of the firing patterns of projection neurons in the cerebral cortex. The balance between excitation and inhibition in the cortical neural circuits is maintained by GABAergic interneurons (Wang et al., 2016). In animals and humans, interneuron loss or malfunctioning can result in mood disorders, working memory disorders, or cognitive decline (Casalia et al., 2021; Williams and Riedemann, 2021), leading to epilepsy (Knopp et al., 2008), Alzheimer’s Disease (AD) (Fu et al., 2017; Najm et al., 2020), schizophrenia (Dienel and Lewis, 2019; Van Derveer et al., 2021), autism spectrum disorder (ASD) (Shin et al., 2021; Deemyad et al., 2022) and other neurological disorders. Researches have revealed that MGE-derived interneuron transplantation offers hope for treating these diseases (De la Cruz et al., 2011; Perez and Lodge, 2013; Upadhya et al., 2019; Lu et al., 2020; Southwell et al., 2020). MGE is a crucial source of inhibitory interneurons during development. The transplanted MGE-derived cells survive and migrate in the cerebral cortex, producing parvalbumin (PV), somatostatin (SST/SOM), and other GABAergic interneuron subtypes, which are integrated into the host circuit, increase inhibitory tension, and regulate neural excitability (Zipancic et al., 2010). Therefore, MGE cells, an excellent source of GABAergic interneurons, play a vital role in the treatment of interneuron disorder-related diseases. In the current review, we discuss the link between interneuron loss or dysfunction and disorders such as epilepsy, schizophrenia, ASD, and AD, and the potential of MGE cells to treat these diseases. The aim is to guide the subsequent transfer of MGE cell transplantation from animal models to clinical application.

## Medial Ganglionic Eminence-Derived Interneurons

The ganglionic eminence (GE) is a temporary brain structure that appears during the middle of embryonic development and is the core region of the ventral brain. The GE includes the MGE, lateral ganglionic eminence (LGE), and caudal ganglionic eminence (CGE). In rodents, the GE gives rise to various neuron types, including projective neurons and interneurons, which distribute widely in the brain (Bandler et al., 2017). The MGE can produce GABAergic interneurons migrating to the striatum and cerebral cortex, GABAergic projection neurons migrating to the globus pallidus, and cholinergic neurons remaining in the basal telencephalon (Marín and Rubenstein, 2001). However, the LGE produces striatal medium spiny neurons and olfactory bulb interneurons. The CGE produces GABAergic projection neurons in the amygdala and other nuclei of the limbic system, and multiple GABAergic interneurons in the cerebral cortex (Wichterle et al., 1999; Eriksson et al., 2003; Zhao et al., 2003; Shi et al., 2021). Most cortical and hippocampal interneurons in mammals are derived from three different regions in the basal telencephalon: the MGE, CGE, and preoptic area (Christodoulou et al., 2021). Approximately 60% of cortical interneurons are derived from the MGE, 30% from the CGE, and the remaining 10% from the preoptic area (Williams and Riedemann, 2021). Inhibitory interneurons play a dominant role in maintaining the functional balance of neural circuits (as elaborated in the next section). Thus, MGE, which provides the most GABAergic interneurons, is critical for nervous system development.

The MGE migrates tangentially from the subpallium to the pallium, producing different GABAergic interneurons in the hippocampus and striatum (Chang et al., 2020). Based on their neurochemical expression profile, GABAergic interneurons can be divided into three categories: PV-positive (PV+), SOM-positive (SOM+)/SST-positive (SST+), and 5-hydroxytryptamine 3A receptor-positive interneurons (Lee et al., 2010; Rudy et al., 2011; Wamsley and Fishell, 2017). The MGE primarily produces cortical interneurons that express PV and SST (Wonders and Anderson, 2006; Bandler et al., 2017; Asgarian et al., 2019). The expression of the transcription factor NKX2.1, in the MGE, is required to specify cortical interneurons expressing PV or SST (Du et al., 2008). Mice with NKX2.1 deletion cannot produce normal MGE tissues (Sussel et al., 1999), or cortical interneurons expressing PV or SST (Xu et al., 2004). PV+ and SST+ interneurons are major contributors to oscillatory activity in the early postnatal hippocampus (Akgul and McBain, 2020). They play an important role in memory function and their defects can lead to related diseases, as described below.

## Interneuron Dysfunction Leads to Related Diseases

The neural network in the vertebrate brain is composed of excitatory neurons and inhibitory interneurons. Although excitatory neurons account for most neural circuits, they only generate a large amount of excitement and cannot perform effective computations. Therefore, the primary inhibitory interneurons play a dominant role in regulating neuronal discharge and maintaining the functional balance of the neural circuits (DeFelipe et al., 2013; Wamsley and Fishell, 2017). Furthermore, interneurons coordinate nervous system activity by producing and stabilizing network oscillations (Benes and Berretta, 2001; Huang et al., 2007). PV+ interneurons rapidly spike and target the initial segment of axons and cell bodies to regulate the discharge of pyramidal cells; interneurons containing SST exhibit slow-firing patterns and synaptic formation in the distal dendrites of pyramidal cells (Perez et al., 2019). Hippocampal PV+ interneurons play a crucial role in generating and maintaining network oscillations associated with learning and episodic memory (Cardin et al., 2009; Sohal et al., 2009). The coordination of these neurons facilitates the synchronization of the activity of complex networks of neurons, which is crucial for maintaining brain function (Benes and Berretta, 2001). The primary task of the interneuron is to provide inhibitory GABAergic synaptic input, a physiological effect of intracellular chloride influx or potassium outflow via homologous GABAA or GABAB receptor activation, respectively, to shunt the membrane away from the action potential threshold and enable instantaneous hyperpolarization (Pelkey et al., 2017). Dysfunction of GABAergic signal transduction, developmental defects, and loss of GABAergic interneurons are related to neurodevelopmental disorders.

Knopp et al. (2008) observed a significant loss of PV− and calmodulin-immunoreactive neurons in pilocarpine-treated mice, resulting in impaired GABAergic inhibition and temporal lobe epilepsy. In mice and patients with AD, tau protein significantly accumulates in GABAergic neurons in the dentate gyrus (DG) of the hippocampus, which inhibits GABAergic signal transmission, impairs hippocampal neurogenesis, and aggravates cognitive impairment in AD (Zheng et al., 2020). Patients and animal models of schizophrenia (Lodge et al., 2009; Kaar et al., 2019) and ASD (Ajram et al., 2017; Paterno et al., 2021) have impaired cortical or hippocampal interneurons and reduced GABAergic inhibition. For example, in patients with schizophrenia, PV expression is reduced in the cortical region, PV and SST expression is reduced in the hippocampus as well (Konradi et al., 2011; Wang et al., 2011; Gonzalez-Burgos et al., 2015). The mechanism by which interneuron dysfunction contributes to these disorders remains unclear. However, interneuron dysfunction, such as GABAergic interneurons, has been linked to several neurological diseases including epilepsy, schizophrenia, ASD, and AD. Supplementing these cells with transplantation strategies is a feasible and effective method to treat related diseases. Since interneuron development is conserved between humans and mice, studying murine interneurons is of interest to understand human biology and disease.

## The Medial Ganglionic Eminence Cell Is a Reliable Source of Cell Therapy

Medial ganglionic eminence cells can survive transplantation into the host, even in harsh environments (Tong et al., 2014; Lu et al., 2020). The study revealed that 1 year after transplantation, the cell survival rate was approximately 20% (Zipancic et al., 2010). Moreover, MGE cells have a strong migration potential. When transplanted into the postnatal brain, embryonic cells from the LGE and dorsal forebrain mostly cluster at the injection site. MGE-derived interneurons are widely dispersed across developmental regions of the central nervous system (CNS), including the striatum, hippocampus, neocortex, amygdala, thalamus, and spinal cord in adults and neonates (Southwell et al., 2014). MGE cells can migrate over long distances within the brain parenchyma, integrate into the host tissue, and differentiate into neurons, including GABAergic neurons (Wichterle et al., 1999). When embryonic MGE cells are transplanted into the neocortex of postnatal mice, MGE cells still follow their inherent migration routes (tangential and radial) and cell fate, survive and integrate into their host circuits (Alvarez-Dolado et al., 2006).

The transplanted MGE-derived cells differentiate into mature cortical interneurons, most of which express GABA, including SST, neuropeptide Y (NPY), PV, and calmodulin subtypes (Alvarez-Dolado et al., 2006). Transplanted MGE cells can modify neural circuits and selectively enhance local inhibition (Alvarez-Dolado et al., 2006). MGE-derived interneurons retain the ability to disperse and integrate functions, receive excitatory and inhibitory inputs, and form inhibitory synapses with host neurons when transplanted into adolescent and adult brains (Wichterle et al., 1999). Thus, MGE cells can replace lost cells and rebuild damaged neural network connections. MGE-derived cell transplantation alters synaptic inhibition and offers a unique opportunity to treat disorders associated with interneuron dysfunction or circuit over-excitation.

Compared to other stem cells, the advantages of MGE cells are as follows: (1) in the adult brain, some neural stem cells are limited in their ability to migrate (Gioia et al., 2020), while MGE cells can migrate widely after transplantation (Tong et al., 2014; Perez et al., 2019; Upadhya et al., 2019); (2) MGE cell transplantation is relatively safe, and teratoma formation has not been observed in related studies of MGE cell transplantation (De la Cruz et al., 2011; Upadhya et al., 2019), while pluripotent stem cells (PSCs) have the risk of tumor formation (Sugaya and Vaidya, 2018); (3) more importantly, most cortical interneurons are generated in the MGE region during development. MGE cells were transplanted into the brain and differentiated into interneurons, expressing GABA and specific interneuron markers, such as PV, SST, NPY, and calretinin (CR). Their ratios were similar to those typically produced by the MGE during development and conform to their intrinsically defined differentiation program (Rodriguez-Martinez et al., 2017). All of these properties make MGE cells the most promising neuronal progenitor cells for cell-based therapies for interneuron disorder-related diseases.

## Potential of Medial Ganglionic Eminence Cell Transplantation for Interneuron Disorder-Related Diseases

### Epilepsy

Epilepsy is a chronic disease in which neurons in the brain fire abnormally, resulting in temporary brain dysfunction. Many studies have revealed that the loss or dysfunction of inhibitory interneurons in the cortex and hippocampus leads to persistent hyperexcitability of neural circuits, resulting in epilepsy (During et al., 1995; Arellano et al., 2004; Knopp et al., 2008). Current anti-epileptic drugs primarily target glutamate receptors or GABA receptors, thereby inhibiting seizures. However, long-term drug therapy may produce severe side effects, and some patients still develop drug resistance (Ghosh et al., 2021). Approximately 30–40% of people with epilepsy are resistant to traditional anti-epileptic drugs (Li et al., 2022). Some of these patients might require surgery to remove epileptic lesions, but even after surgery, some patients have recurrent seizures and a range of neurological deficits (Sinha et al., 2021). Therefore, a safe and effective therapeutic strategy must be developed. In the CNS, GABA-mediated inhibition is crucial for controlling synaptic circuits and inhibiting neuronal circuitry overexcitation, preventing the spread of epileptic discharges (Freund and Buzsáki, 1996). Several subpopulations of interneurons, including dendritic cells expressing PV (Gallo et al., 2020), basket cells expressing SST (Baraban, 1998), and NPY (Baraban, 1998) have been associated with the suppression of seizures. Therefore, inhibitory interneuron transplantation is a promising therapeutic option for epilepsy.

#### The Efficiency of Medial Ganglionic Eminence Cell Transplantation as a Prophylactic Treatment

Initial investigations on the efficacy of MGE progenitor cells for alleviating seizures were mostly prophylactic. Before induction of an animal model of epilepsy, MGE cells were transplanted into the cerebral cortex, hippocampus, and other regions. In these studies, spontaneous seizures were induced by maximal electroshock (Calcagnotto et al., 2010), local injection of pentylenetetrazol (Zipancic et al., 2010), or 4-aminopyridine (4-AP) (De la Cruz et al., 2011). Shetty and Upadhya (2016) reviewed partially relevant studies before 2011, which are not described here. In a subsequent study, cells from the MGE region of embryonic day (E) 13.5 mice were injected into the sensorimotor cortex of adult CD1 mice aged 6–8 weeks (De la Cruz et al., 2011, Table 1). Acute focal epileptic discharge was induced by injecting 4-AP, 2 mm from the graft site at 1, 2.5, and 6–8 weeks after cell transplantation. One day after transplantation, De la Cruz et al. (2011) observed that MGE-derived progenitor cells into layers 2–6 of the mouse cortex, and a lot of cells near the injection site displayed bipolar morphology typical of migrating interneurons. Immunofluorescence detected the expression of several markers of cortical interneuron 2 months after transplantation. MGE cells significantly reduced the 4-AP-induced discharge power 2.5 or 6–8 weeks after transplantation compared to the dead MGE cells of the control group. This study demonstrated that MGE cell transplantation alleviated seizures independent of the number of transplanted cells. Additionally, 1 day after transplantation dual immunofluorescence for green fluorescent protein (GFP) and Ki-67 was rare, indicating that only a small proportion of MGE cells were still undergoing cell division in the adult brain 24 h after transplantation. No tumors or cysts were observed in this study by De la Cruz et al. (2011). Prophylactic studies have shown preliminary promise for treating epilepsy using MGE cells.

**TABLE 1:** Medial ganglionic eminence (MGE) cell transplantation for epilepsy.

Study	Animal model	Cell type and quantity of transplantation	Time and site of transplantation	Differentiation into interneurons	Effects of grafts on host brain changes	Effects on neural function
De la Cruz et al., 2011	CD1 mice/epilepsy induced by 4-aminopyridine	Cells from the MGE of E13.5 mice embryos/1.5 × 10^4^ cells	8–10 weeks old CD1 mice/sensorimotor cortex	SST (dMGE: 39.54 ± 3.80%; vMGE: 38.29 ± 4.86%). PV (dMGE: 12.49 ± 2.73%; vMGE: 16.59 ± 3.89%).	MGE cells survive and migrate.	Reduce the power of 4-aminopyridine induced discharges.
Romariz et al., 2017	Male SD rats/epilepsy induced by pilocarpine	Freshly MGE cells from E14.5 SD rat and MGE cell harvested as neurospheres/4 × 10^5^ cells.	7–10 days after SE/bilateral hippocampus.	The Pilo-FR group: NPY (12.38%), PV (6.96%), CR (5.94%); the Pilo-WD-RA group: NPY (1.52%), PV (1.89%), CR (3.4%); the Pilo-GF group: NPY (1.87%), PV (2.43%), CR (0.69%).	Fresh MGE cells produce more inhibitory interneurons.	Fresh MGE cells show stronger anticonvulsant effects.
Gupta et al., 2019	Male C57BL/6NHsd mice/TLE induced by pilocarpine	Cells from the MGE of E13.5 transgenic mouse embryos/1 × 10^5^ cells per a single injection site	2 weeks after SE/four sites in each hippocampus	PV (37.3%); SOM (33.8%); nNOS (12.4%); CR (7.9%)	(1) Form functional inhibitory connections with adult born granule cells; (2) Limit distal dendritic growth in hippocampal dentate granule cells.	–
Casalia et al., 2017	Male CD1 mice/epilepsy induced by pilocarpine	Cells from the MGE of E13.5 β-actin GFP mice/3 × 10^4^ cells per injection.	2 months after SE/bilateral hippocampus.	PV; SOM; nNOS	MGE cells migrate and differentiate.	(1) Save behavioral complications. (2) Suppress epileptic activity.
Upadhya et al., 2019	Male F344 rats/SE induced by kainic acid	hiPSC-derived MGE cells/3 × 10^5^ cells	7 days after SE/bilateral hippocampus	PV (27%); NPY (11%); SST (6%); CR (8%)	(1) Grafts survive and migrate extensively. (2) Reduce abnormal mossy fiber germination and abnormal neurogenesis in the dentate gyrus. (3) Synapses form between graft-derived axons and excitatory neurons of the host.	(1) Inhibit spontaneous recurrent seizures in the chronic phase and decrease EEG power. (2) Improve the cognitive and pattern separation dysfunction.

*MGE, medial ganglionic eminence; E, embryonic day; SST/SOM, somatostatin; dMGE, dorsal MGE; vMGE, ventral MGE; PV, parvalbumin; SD, Sprague-Dawley; SE, status epilepticus; the Pilo-FR group, epileptic rats that received freshly isolated MGE cells; NPY, neuropeptide Y; the Pilo-WD-RA group and Pilo-GF group, epileptic rats that transplanted MGE cells cultured as neurospheres in the WD-RA, and GF conditions; CR, calretinin; TLE, temporal lobe epilepsy; nNOS, neuronal nitric oxide synthase; GFP, green fluorescent protein; hiPSC, human-induced pluripotent stem cell; EEG, electroencephalograph.*

#### The Influence of Medial Ganglionic Eminence Cell Transplantation After the Occurrence of Epilepsy

Studies have revealed that injecting MGE cells into the brains of epileptic animal models, such as voltage-gated potassium channel 1.1 -/- animal (Baraban et al., 2009) and Stargazer mouse model of absence epilepsy (Hammad et al., 2015), significantly reduced the number of seizures and the mortality of epilepsy models. Transplantation of MGE cells is a potential clinical treatment for epilepsy.

New progress has been made in the treatment of epilepsy using MGE cells. One study (Romariz et al., 2017, Table 1) compared the anticonvulsant effects and cell differentiation patterns of neurosphere−grown MGE cells and freshly isolated MGE cells. MGE region of E14.5 Sprague Dawley rats was dissected to harvest cells. Cells were transplanted into the hippocampus 7–10 days after the development of status epilepticus (SE). Transplantation of fresh MGE cells and neurosphere−cultivated MGE cells in the presence of growth factors reduced the number of seizures. Their mechanisms are slightly different: fresh MGE cells preferentially differentiate into inhibitory neurons to regulate neural activity and alleviate seizures, while MGE cells cultivated as neurospheres under growth factor conditions may modulate the frequency of epileptic seizures through glia-related mechanisms, such as secretion of nutrient factors and regulation of inflammation. However, transplantation of fresh MGE progenitor cells exhibited more potent anticonvulsant effects than transplantation of neurosphere−grown MGE cells. The possible reasons may be as follows: (1) fresh MGE cells have a regional molecular memory and can differentiate into inhibitory interneurons *in vivo* after transplantation; (2) culture medium molecules influence the fate and differentiation of MGE cells as nerve spheres. Therefore, freshly obtained MGE cells may be more suitable for transplantation. Another possible structural mechanism for the anti-epileptic effect of MGE cells is increased GABAergic innervation by transplanted MGE progenitor cells, which may limit dendritic growth in adult hippocampal dentate granule cells (Gupta et al., 2019, Table 1). MGE progenitor cells from E13.5 GFP mice were transplanted into the hippocampus of mice 2 months after SE (Casalia et al., 2017, Table 1). At 210 days after transplantation, the grafts differentiated into multiple GABAergic interneuron subtypes, including PV+ and SST+ interneuron. Similar expression patterns were still detected 12 months after transplantation, confirming the long-term integration of MGE-derived interneurons in the mouse brain.

Fetal MGE cells have a potential positive value in epilepsy applications. Nevertheless, to a certain extent, ethical issues and xenograft rejection limit its transformation into the clinic. A more appropriate method is needed, and human-induced PSC (hiPSC)-derived MGE neural progenitor cells are an excellent choice. A 2019 study explored this option (Upadhya et al., 2019, Table 1). MGE-like neural progenitor cells derived from hiPSCs were transplanted into the hippocampus of rats 7 days after SE. Histological results showed that the transplanted cells survived well, and most differentiated into GABAergic interneurons (76%), including PV+ (27%) and NPY-positive (NPY+) (11%) interneuron subtypes. More importantly, synapses formed between graft-derived axons and excitatory neurons of the host. Continuous video electroencephalograph (EEG) recordings showed that the frequency and severity of spontaneous recurrent seizures in rats receiving bilateral hiPSC-MGE cell transplantation were significantly reduced. In addition, MGE cell transplantation can improve cognitive and pattern separation dysfunction in epileptic rats to a certain extent. MGE cells were transplanted into the hippocampus, migrated to the cell layer of the hippocampus, including DG, CA1 subfield, and CA3 subfield, and differentiated into GABAergic interneurons, which then enhanced functional synaptic inhibition, decreased nerve excitability, and reduced abnormal mossy fiber germination and abnormal neurogenesis in the DG, which provided evidence for the amelioration of behavioral defects in epileptic rats. Excitingly, no teratoma formation was observed 5 months after transplantation.

As an essential source of GABAergic interneurons in the cerebral cortex and hippocampus, MGE cells are ideal for cell transplantation. The transplanted MGE cells not only differentiate into GABAergic interneurons but also form inhibitory synapses and increase the transmission of GABAergic synapses to adjacent pyramidal cells (Alvarez-Dolado et al., 2006). Cell replacement therapy restores the excitatory-inhibitory imbalance, which may be central to treating many forms of epilepsy. Over the past decade, MGE cell transplantation has shown remarkable success in the prevention and treatment of epilepsy.

### Schizophrenia

Schizophrenia is an unknown etiology of heterogenous psychosis, which can manifest with personal sensory abnormalities along with emotional and behavioral abnormalities. The core characteristics are positive symptoms (delusions and hallucinations; so-called psychotic symptoms of loss of connection with reality), negative symptoms (impaired motivation, spontaneous speech loss, and social withdrawal), and further cognitive impairment (Jauhar et al., 2022). The exact pathophysiological mechanism has not yet been elucidated, and the dopamine hypothesis (that the mesolimbic dopamine system is overactive) is widely supported (Jauhar et al., 2022). However, there is no direct evidence of primary changes in dopamine neurons (Harrison, 1999). The main antipsychotic drugs, which function by blocking dopamine receptors, reduce symptoms and cause severe side effects, such as Parkinson’s extrapyramidal symptoms and tardive dyskinesia (Jauhar et al., 2022). Several other treatments, including psychotherapy, cognitive-behavioral therapy, and electric shocks, have been unsuccessful. It has been hypothesized that GABA dysfunction plays a central role in the pathophysiology of schizophrenia (Roberts, 1972). Selective deletions of PV and SST expression in the hippocampus and cortex have been found in post-mortem brain tissue (Perez et al., 2019). PV+ and SST+ interneurons are essential for the stability of neural network activity and the coordination of brain oscillations (Medrano-Fernandez et al., 2019). In particular, PV+ interneurons play an important role in the inhibitory control of pyramidal cell activity (Lodge et al., 2009). Hyperactivity in the resting hippocampus has been observed in patients and rodent models with schizophrenia (Lodge and Grace, 2007; Schobel et al., 2009). Symptoms associated with schizophrenia are caused by abnormalities in the function of the downstream dopaminergic system when hippocampal activity increases (Donegan et al., 2017). Therefore, restoring GABAergic inhibitory interneurons may be a promising strategy for the treatment of schizophrenia.

In a study conducted in 2013, cells from the MGE region of the gestational day 14.5 GFP-positive (GFP+) transgenic rats were transplanted into the hippocampus of methylazoxymethanol acetate (MAM)-treated rats (40–45 days old) (Perez and Lodge, 2013, Table 2). Two months after transplantation, the cells migrated to the entire hippocampus and the adjacent areas. Immunostaining showed that 18.1% of GFP+ cells were PV+. Normalization of the enhanced ventral hippocampus discharge rate of pyramidal neurons was observed in MAM-treated rats after MGE cell transplantation. Furthermore, the improved motor response to low amphetamine doses was reversed after transplantation. This study demonstrated that MGE cells, transplanted into the ventral hippocampus in MAM-treated rats, normalized abnormal pyramidal neuronal activity and downstream dopamine system function and had a potential therapeutic effect on the positive symptoms of schizophrenia. A subsequent study transplanted cells from the MGE region of E15.5 mice into the hippocampus of adult cyclin dopamine receptor 2 knockout (Ccnd2 KO) mice (6–8 weeks old) and yielded equivalent results (Gilani et al., 2014, Table 2). Immunolabeling indicated that 97% of the GFP+ cells were GABAergic, 56% expressed PV, and 35% expressed SST. In addition to normalizing hippocampal metabolic activity and dopaminergic neural activity, such transplantation also improves cognitive function in mice. A recent study of the lysophosphatidic acid receptor 1 deficient mice led to hippocampal and behavioral abnormalities that mimic the core features observed in common neuropsychiatric disorders (Rosell-Valle et al., 2021). MGE cell transplantation improved behavioral defects in mice and compensated for the loss of interneurons.

**TABLE 2:** Medial ganglionic eminence cell transplantation for schizophrenia.

Study	Animal model	Cell type and quantity of transplantation	Time and site of transplantation	Differentiation into interneurons	Effects of grafts on host brain changes	Effects on neural function
Perez and Lodge, 2013	Male SD rats/MAM model of schizophrenia	Cells from MGE-derived tissue of gestational day 14.5 GFP transgenic rats/-	40–45 days old MAM-treated rats/bilateral ventral hippocampus	PV (18.1%)	(1) Normalize abnormal pyramidal neuronal activity. (2) Downstream dopamine system function.	Have a potential therapeutic effect on positive symptoms of schizophrenia
Gilani et al., 2014	Cyclin dopamine receptor 2 knockout mice	Cells from the MGE of E15.5 GFP transgenic mice/3 × 10^4^ live cells per microliter	6–8 weeks old cyclin dopamine receptor 2 knockout mice/CA1 area of the bilateral caudoventral hippocampus	PV (56%); SST (35%)	Differentiate into GABAergic interneurons, which are distributed in the whole longitudinal axis of the hippocampus.	(1) Decrease hippocampal metabolic activity; (2) Improve context-dependent learning and memory; (3) Normalize dopamine neuronal activity and behavioral response to amphetamines

*MAM, methylazoxymethanol acetate.*

These studies illustrate the potential value of MGE cells, which produce many GABAergic interneurons, for the treatment of schizophrenia. In the future, we can further explore the exact mechanism of MGE cell transplantation in the treatment of schizophrenia, including whether the secretion of neurotrophic factors and hippocampal neurogenesis is involved.

### Autism Spectrum Disorder

Autism spectrum disorder is an increasingly common neurodevelopmental disorder characterized by problems with social interaction, communication, and repetitive and stereotypical behaviors (Bremer et al., 2016). The etiology is still not fully understood, and it is generally believed that it may be related to genetics, infection, immunity, and physicochemical factors during pregnancy (Mandy and Lai, 2016; Martinez-Cerdeno et al., 2016). Currently, there is no specific drug for the core symptoms of ASD, only for symptomatic treatment (Ajram et al., 2017). An imbalance between excitation and inhibition may be the neurobiological basis of ASD (Bozzi et al., 2018). There is evidence that the disruption of GABAergic signaling may be the underlying mechanism leading to the development of autism (LeBlanc and Fagiolini, 2011). Studies in several mouse models of ASD and patients with ASD further support the hypothesis of GABAergic interneuron disorders, particularly PV+ interneuron loss (Ajram et al., 2017; Hashemi et al., 2017; Vogt et al., 2018; Di et al., 2020; Paterno et al., 2021). PV+ interneurons are fast spike cells that synchronize the activity of pyramidal cells through peri-somatic and axo-axonic inhibition. Reduced numbers of PV+ interneurons may disrupt the excitatory-inhibitory balance and alter gamma wave oscillations in the cortex of autistic patients (Hashemi et al., 2017). Therefore, restoring the interneurons in specific areas may be an effective therapeutic approach.

A previous study of embryonic stem cell (ESC)-derived interneurons transplanted into GABA-deficient ASD models demonstrated that PV+ interneuron transplantation reduced pyramidal neuron firing in the prefrontal cortex (PFC) and ameliorated deficits in social interaction and cognitive flexibility (Donegan et al., 2018). This study suggested that enhanced GABA inhibition was associated with improved behavioral deficits in ASD. Does interneuron transplantation also improve the related behavioral deficits in mouse models of ASD with excessive synaptic inhibition? Phosphatase *Pten* mutant mice exhibited deficits in social behavior, increased synaptic inhibition in the PFC, altered composition of cortical interneuron subtypes, and abnormal EEG baseline (Vogt et al., 2015; Southwell et al., 2020). MGE cells from E13.5 mice expressing GFP were injected into the bilateral PFC of neonatal *Pten* conditional knock-out (cKO) recipient mice in research by Southwell et al. (2020, Table 3). Seven weeks after transplantation, the behavioral analysis revealed a significant increase in social activity in mice, while patch-clamp recordings indicated increased spontaneous inhibitory postsynaptic current frequency in pyramid-based neurons. Eight weeks after transplantation, MGE-derived interneurons expressed PV, SST, and Reelin markers. The following results were observed: (1) MGE cell therapy could salvage the behavioral deficits of ASD even in the context of over-inhibition rather than a lack of inhibition; (2) MGE cell therapy could improve behavioral deficits without normalizing synaptic inhibition levels or EEG baseline. Therefore, transplantation may rescue autistic behaviors not only by simply normalizing pathological neural circuits but also by inducing new physiological states to produce therapeutic effects, which requires further exploration.

**TABLE 3:** Medial ganglionic eminence cell transplantation for autism spectrum disorder and Alzheimer’s Disease.

Study	Animal model	Cell type and quantity of transplantation	Time and site of transplantation	Differentiation into interneurons	Effects of grafts on host brain changes	Effects on neural function
Southwell et al., 2020	Pten conditional knock-out recipient mice	MGE cells from E13.5 heterozygous GFP embryos/(8 × 10^4^ – 1 × 10^5^) cells per hemisphere	Neonatal Pten conditional knock-out recipient mice/bilateral prefrontal cortex	SST (16.3 ± 3.9%); PV (24.4 ± 3.5%); Reelin (10.1 ± 1.8%)	Fail to normalize synaptic inhibition levels or EEG baseline	Salvage the behavioral deficits of autism spectrum disorder even in the context of over-inhibition rather than lack of inhibition
Tong et al., 2014	Female ApoE3-KI and ApoE4-KI homozygous mice.	MGE cells from E13.5 enhanced GFP mice embryos/2 × 10^4^ cells at each site	14-month-old ApoE4-Ki mice/bilateral hilus	SOM (E4-KI:45.7 ± 4.4%; E3-KI:45.0 ± 5.3%); PV (E4-KI:12.5 ± 2.4%; E3-KI:12.1 ± 2.6%); NPY (E4-KI:23.0 ± 4.1%; E3-KI:23.8 ± 3.5%)	Mature and integrate into host neural circuits	Improve cognitive deficits in Alzheimer’s Disease mice in the presence of amyloid-β accumulation
Lu et al., 2020	Male APP/PS1 transgenic mice	Cells from the MGE of E13.5 enhanced GFP transgenic mice/2 × 10^4^ cells each site	7-month-old APP/PS1 mice/bilateral dentate gyrus of the hippocampus	NPY (16.850 ± 1.438%); CR (24.117 ± 2.541%); PV (27.783 ± 3.948%)	No effect on accumulated amyloid-β in APP/PS1 mice	(1) Reduce hyperexcitability. (2) Improve the suppression of long-term potentiation and synaptic plasticity, (3) Rescue cognitive deficits

*ApoE3-KI, ApoE3 knock-in; ApoE4-KI, ApoE4 knock-in; APP, amyloid precursor protein; PS1, presenilin 1.*

### Alzheimer’s Disease

The balance between excitatory and inhibitory neural networks is essential for maintaining normal brain function, especially cognitive function (Vico Varela et al., 2019). An excitatory-inhibitory imbalance can lead to neurological disorders such as AD. AD is a central neurodegenerative disease characterized by progressive cognitive dysfunction, and its pathogenesis is complex. The amyloid precursor protein (APP) and presenilin (PS) 1 or PS2 are thought to be involved in the development of early onset AD. They promote the aggregation and accumulation of amyloid-β (Aβ) in the brain, and the accumulation of oligomeric Aβ leads to tau hyperphosphorylation (Huang and Mucke, 2012). The loss of GABAergic neurons in mice with early Aβ deposition begins at 6 months. Additionally, glutamic acid decarboxylase (GAD), SOM, and PV are significantly reduced in the hippocampus of mice with high expression of human tau protein. Therefore, the pathological markers of AD may be related to impaired GABA signal transmission (Jimenez-Balado and Eich, 2021). Apolipoprotein E4 (ApoE4) may induce GABAergic interneuron injury through both Aβ-dependent and -independent effects, resulting in reduced neurogenesis in the hippocampus and impaired learning and memory (Huang and Mucke, 2012). The loss of GABA may be the trigger factor leading to the over-excitation of neural network activity in AD, and over-excitation of the neural network is one of the reasons leading to cognitive impairment in AD patients. AD is difficult to be identified in an early stage. Most cases are identified in the middle and late stages of development. Therefore, the treatment of middle- and late-stage AD is critical. However, therapies targeting specific AD-related pathways have failed in human trials of advanced AD (Huang and Mucke, 2012). Patients with advanced AD are no longer treated with existing drugs, due to the loss of inhibitory interneurons in the hippocampus. Inhibition of hyperexcitability has been shown to ameliorate cognitive deficits in AD (Haberman et al., 2017). Cortical GABAergic interneurons are produced in embryonic MGE. MGE cells can survive, migrate, and differentiate into a variety of inhibitory interneurons when transplanted into rodent hippocampi. MGE cells are widely used in the study of neurological diseases, such as epilepsy (Casalia et al., 2017; Romariz et al., 2017; Backofen-Wehrhahn et al., 2018; Gupta et al., 2019; Upadhya et al., 2019), and schizophrenia (Perez and Lodge, 2013; Gilani et al., 2014). Therefore, transplanting MGE neural progenitor cells into the hippocampus of patients with AD may offer new insights.

The hippocampal hilus of 14-month-old female ApoE4 knock-in mice was injected with a suspension of fresh cells from the MGE region of E13.5 mice (Tong et al., 2014, Table 3). The transplanted cells migrated throughout the hilus, with the neurites extending to the molecular layer of the DG, survived (>3 months), and differentiated into mature GABA-inhibitory interneurons (96.1 ± 2.1%), including SOM+, NPY+, and PV+ interneurons. MGE cell transplantation increased the frequency of spontaneous inhibitory currents and increased inhibitory charge transfer in the dentate granule neurons. Electrophysiology verified that MGE-derived GABAergic interneurons were functionally integrated into the host hippocampal circuitry. MGE cell transplantation also improved cognitive deficits in AD mice in the presence of Aβ accumulation. In a 2020 study, similar changes were observed in the bilateral hippocampal DG of 7-month-old APP/PS1 mice transplanted with MGE cells from E13.5 enhanced GFP transgenic mice (Lu et al., 2020, Table 3). APP/PSL mice, a mature and widely used AD model, overexpress the human APP and PS1, leading to elevated levels of Aβ42 (Hollnagel et al., 2019). APP/PS1 mice showed reduced hippocampal CR positive (CR+) and NPY+ interneurons at both 7 and 12 months, and reduced PV+ interneurons at 12 months (Lu et al., 2020, Table 3). These results indicate an age-dependent loss of GABAergic interneurons associated with AD pathology and cognitive deficits. On the 30 days after transplantation, the transplanted cells have migrated to the DG subregion, and a minority of cells have migrated outside the DG subregion. On the 60 days after transplantation, MGE cells showed good migration in the hippocampus and differentiated into GABA interneurons and their subtypes, including NPY+, CR+, and PV+ interneurons. EEG monitoring suggested that MGE cell transplantation reduced hyperexcitability in the APP/PS1 transgenic mice. Additionally, improved learning and memory function in APP/PS1 mice were further verified by behavioral tests. MGE cell transplantation also improved long-term potentiation and synaptic plasticity. However, no effect on Aβ was observed in these two studies by Tong et al. (2014) and Lu et al. (2020), which may be related to the time point of cell transplantation. In conclusion, the above studies indicate that MGE cells can still differentiate into neurons and restore the normal cognitive function of AD even in harsh environments, which has immense potential in treating AD. The mechanism may be related to the excitatory-inhibitory regulation of neural network activity, but whether other mechanisms are involved requires further investigation.

## Conclusion

Medial ganglionic eminence cells are a valuable source for the treatment of various neurological diseases. In addition to these diseases mentioned above, MGE cell transplantation can improve memory impairment after traumatic brain injury (Zhu et al., 2019), neuropathic pain after spinal cord injury (Fandel et al., 2016), and motor function in rats with Parkinson’s disease (Martínez-Cerdeño et al., 2010). MGE cells are a viable potential treatment for diseases associated with interneuron disorders based on the following points: (1) MGE cells are transplanted into the host brain for long-distance migration and differentiation into mature interneurons; (2) interneurons derived from transplanted cells establish synaptic connections with the host neurons and selectively modify neural circuits to enhance inhibition; (3) MGE cell therapy can reduce mortality in animals with partial genetic defects; (4) MGE cell therapy improves cognitive and mood disorders.

Genetic variation is an important factor that leads to the occurrence of diseases associated with interneuron disorders. With an in-depth understanding of the pathogenesis of diseases, other cutting-edge approaches, including gene therapy and re-programming, have emerged. This provides a new direction for the treatment of CNS diseases by upregulating or knocking down specific pathogenic genes or inducing human non-neuron cells to transform into neurons to replace damaged neurons. Compared with them, the advantages of MGE cell transplantation are as follows: (1) safety: cells are directly injected into the target area for cell transplantation without vectors; however, gene therapy or re-programming may require the use of viral vectors, which may cause the body to initiate an immune response, activate macrophages (Zhang and Wang, 2021), and there is a risk of off-target (Pattanayak et al., 2013; Turner et al., 2021); (2) influence of intracerebral environment on differentiation pattern: MGE cells are transplanted to the specific damaged brain area, follow their inherent migration routes and cell fate, differentiate into interneurons, form functional synapses, and reconstruct neural circuits (Zipancic et al., 2010); the efficiency and subtype specifications of *in vivo* re-programming may be influenced by environmental signals after brain injury (Vignoles et al., 2019); (3) stable internal environment: Grafts secrete neuroprotective factors and release factors that repair and fight inflammation and scars to create a homeostasis environment (Chohan and Moore, 2016).

However, some problems remain to be solved before MGE cell transplantation can be safely used clinically. First, there is the problem of cell origin. Currently, most MGE cells used in animal experiments are derived from the MGE region of fetal mice. Due to ethical problems, it is impossible to obtain MGE cells from human fetuses for clinical application. Therefore, it is important to generate similar MGE cells through directed differentiation of human PSCs (hPSCs) (including hiPSCs and human ESCs). There is no lack of research on the generation of MGE-like neural progenitor cells from hPSCs, which then differentiate into GABAergic interneurons and mature *in vitro* (Liu et al., 2013; Noakes et al., 2019; Li et al., 2021). Ground-breaking researches have shown that MGE cells derived from hPSCs can be used for the treatment of epilepsy (Upadhya et al., 2019), neuropathic pain (Fandel et al., 2016), and stroke (Li et al., 2021), opening the possibility of developing suitable human therapies. If the efficiency of inducing differentiation can be improved and large-scale amplification of functional MGE progenitor cells can be achieved, this will greatly improve the accessibility of these cells (Ma et al., 2019). In this process, attention must also be paid to the safety of hPSC-derived progenitor cells to avoid the formation of tumors. Second, further studies are needed to explore the underlying mechanisms of interneuron disorder-related diseases, to promote the differentiation of these transplanted cells into appropriate interneuron subtypes and their integration into local circuits for long-term efficacy. Third, transplantation is an invasive intervention, and the choice of transplantation route requires more consideration. Transplantation of stem cells intravenously was thought to reduce invasiveness (Chu et al., 2004). However, most of the stem cells remained in the lungs as they circulated and were cleared by the body (Pendharkar et al., 2010). Even when they reached the brain, only 10% of the cells differentiated into neurons (Chu et al., 2004). Therefore, transplantation of cells intravenously may not be the ideal route for MGE cells. A large number of studies on MGE cell transplantation were performed by intracerebral injection (Calcagnotto et al., 2010; Gupta et al., 2019; Upadhya et al., 2019). Intracerebral injection of MGE cells can directly target the desired graft region, reduce cell loss during long-term migration, and more cells can differentiate into neurons compared to transplantation of cells intravenously. Finally, although intracerebral transplantation can transport cells directly to the target area, it is an invasive intervention and therefore strict use conditions should be set, which may limit its clinical application. In cases of drug resistance, severe drug side effects, recurrent attacks, or ineffective surgical treatment, options can be made after weighing the pro and cons in the future. There is still a long way to go before MGE cell therapy for neuropsychiatric diseases can be translated into clinical practice. However, there are reasons for optimism, given the growing research into the mechanisms of interneuron disorder-related diseases and the rapid development of cell differentiation techniques.

## Author Contributions

XH conceived and revised the manuscript. DL designed and drafted the manuscript. QW collected literatures. All authors contributed to the article and approved the submitted version.

## Conflict of Interest

The authors declare that the research was conducted in the absence of any commercial or financial relationships that could be construed as a potential conflict of interest.

## Publisher’s Note

All claims expressed in this article are solely those of the authors and do not necessarily represent those of their affiliated organizations, or those of the publisher, the editors and the reviewers. Any product that may be evaluated in this article, or claim that may be made by its manufacturer, is not guaranteed or endorsed by the publisher.
